# Preparation, Characterization, and Inhibition of Hyaluronic Acid Oligosaccharides in Triple-Negative Breast Cancer

**DOI:** 10.3390/biom9090436

**Published:** 2019-09-01

**Authors:** Wenwei Han, Lili Song, Yingdi Wang, Youjing Lv, Xiangyan Chen, Xia Zhao

**Affiliations:** 1 Key Laboratory of Marine Drugs, Ministry of Education, Shandong Provincial Key Laboratory of Glycoscience and Glycoengineering, School of Medicine and Pharmacy, Ocean University of China, Qingdao 266003, China; 2 Laboratory for Marine Drugs and Bioproducts of Qingdao National Laboratory for Marine Science and Technology, Qingdao 266237, China; 3 Marine Biomedical Research Institute of Qingdao, Qingdao 266071, China

**Keywords:** hyaluronic acid, oligosaccharides, preparation, structural characterization, triple-negative breast cancer

## Abstract

Hyaluronic acid (hyaluronan, HA) is a critical component of the extracellular matrix and plays an important biological function of interacting with different molecules and receptors. In this study, both odd- and even-numbered HA oligosaccharides (HAOs) with specific degrees of polymerization (DP) were prepared by different hydrochloric acid hydrolyses, and their structures were characterized by means of HPLC, ESI-MS, and NMR. The data show that the odd-numbered HAOs (DP3-11) have a glucuronic acid reducing end, while the even-numbered HAOs (DP2-10) have an *N*-acetylglucosamine reducing end. Biological evaluations indicated that all HAOs significantly inhibited the growth and migration of triple-negative breast cancer (TNBC) MDA-MB-231 cells. Among these oligosaccharides, the HA tetrasaccharide (DP4) was confirmed to be the minimum fragment necessary to inhibit MDA-MB-231 cells. Our data suggest that HAOs have potential value in the treatment of TNBC.

## 1. Introduction

Hyaluronic acid (hyaluronan, HA) is a major component of the extracellular matrix and is a nonsulfated glycosaminoglycan composed of β-*N*-acetylglucosamine (GlcNAc)-(1,3)-β-glucuronic acid (GlcA)-(1,4) -repeating units. Accumulating evidence suggests that HA is involved in a variety of cellular processes, including cell adhesion, migration, and proliferation [1,2]. HA was reported to have interacted with various cell surface receptors such as receptors for HA-mediated motility (RHAMM) [3], cluster of differentiation 44 (CD44) [4,5,6], lymphatic vessel endothelial hyaluronan receptor (LYVE1) [7], and toll-like receptors (TLRs) [8]. Recent studies have provided evidence that HA plays an important role in cancer initiation, proliferation, metastasis, and progression [9,10,11]. 

Breast cancer is the leading type of cancer in women worldwide, accounting for 25% of all cases [12,13]. Triple-negative breast cancer (TNBC) is a high-grade and aggressive breast cancer and often results in high patient mortality because these cancer cells do not express the estrogen receptor, progesterone receptor, and human epidermal growth factor receptor 2 (HER2) [14]. Cell culture studies showed that the invasive breast cancer cells synthesize and accumulate larger amounts of HA than normal tissue, especially remarkable in TNBC cells [15]. A number of studies have demonstrated that HA regulates tumor cell growth and invasion in vitro and in vivo [16,17,18]. Over-accumulation of high molecular weight HA (HMW-HA) enhances the growth and migration of tumors in mammary epithelial cells of MMTV-Neu transgenic mice [19]. Furthermore, a large amount of HMW-HA has been reported to promote breast cancer cell invasion in vitro [20]. These results demonstrate that increased HA in the tumor microenvironment supports neoplastic progression.

Accumulating evidence suggests that HA oligosaccharides (HAOs) display unique biologic activities that are not shared by native HA, and HAOs show different bioactivity compared to HA during cancer progression. HA oligomers have been reported to inhibit tumor growth in vivo [21]. HA oligomers digested by hyaluronidase can localize breast tumor growth and prevent invasive spread, which is opposite to the effects of HMW-HA polymers [22]. However, the compositions and structures of these HA fragments still remain unclear. Because of the lack of information, it is difficult to develop the relationship between HA fragments and pathological conditions. Therefore, a better understanding of the interaction between well-characterized HAOs and TNBC is highly warranted and may lead to improve breast cancer treatment. Thus, we try to explore whether HAOs can interfere with the growth and migration of TNBC. 

Here, we prepared both odd- and even-numbered HAOs with specific DP. They were well-characterized, and their interactions with MDA-MB-231 cells were studied. 

## 2. Materials and Methods

### 2.1. Materials and Reagents

HA fine powder (~40 kDa) was purchased from Bloomage Freda Biopharm (Jinan, China). Resazurin sodium salt and fluorescein isothiocyanate (FITC) were purchased from Sigma-Aldrich (Saint Louis, Missouri, MO, USA). 1-Phenyl-3-methyl-5-pyrazolone (PMP) was purchased from Aladdin (Shanghai, China). All other chemicals and solvents used were of analytical grade unless otherwise specified. 

MDA-MB-231 TNBC cells were obtained from the Cell Bank of the Chinese Academy of Sciences (Shanghai, China) and were maintained in Dulbecco’s modified Eagle medium (DMEM) supplemented with 10% fetal bovine serum (FBS), 100 U per mL of penicillin, and 100 μg/mL of streptomycin. Cells were incubated at 37 °C in an atmosphere of 5% vol. CO_2_. Cell culture was limited to seven passages.

### 2.2. Preparation of Oligosaccharides from Hyaluronic Acid (HA)

HA (100 mg) was suspended in 1.0 mol/L HCl aqueous solution (10.0 mL). After stirring at 90 °C for 15 min, the reaction solution was neutralized to pH 7.0 by dripping 1.0 mol/L NaOH aqueous solution. Subsequent freeze-drying gave HA oligosaccharide 1 (HAO1). HA (100 mg) was suspended in 0.1 mol/L HCl aqueous solution (10.0 mL). After stirring at 90 °C for 20 min, the reaction was neutralized by 1.0 mol/L NaOH aqueous solution. Subsequent freeze-drying gave HA oligosaccharide 2 (HAO2). HAO1 and HAO2 were fractionated by gel exclusion chromatography (GPC) on a Bio-Gel P10 column (500 mL, 2.6 × 100 cm, Bio-Rad Laboratories, Hercules, CA, USA). NH_4_HCO_3_ aqueous solution (0.1 mol/L) was used as mobile phase at a flow rate of 0.5 mL/min. Then, HAOs with specific degrees of polymerization (DP) were obtained.

### 2.3. HPLC Analysis of HA Oligosaccharides

High-performance liquid chromatography (HPLC) analysis of HAOs was performed on a BioBasic SE-120 column (5 µm, 7.8 × 300 mm). Na_2_SO_4_ aqueous solution (0.1 mol/L) was used as mobile phase at a flow rate of 1.0 mL/min. The concentration of sample was 5 mg/mL, and the column temperature was kept at 35 °C.

### 2.4. Thin Layer Chromatography of HA Oligosaccharides

Thin layer chromatography (TLC) was performed on a silica gel plate 60 F254 (Merck KGaA, Darmstadt, Germany). Plates were developed using *N*-butanol/water/formic acid solvent (4/1/6, respectively). The staining solution was described by Robyt [23]. Spots were visualized by heating at 250 °C for 20–30 s. The retardation factor (Rf) was calculated as the ratio of the distance traveled by the center of a spot to the distance traveled by the solvent front.

### 2.5. Electrospray Mass Spectroscopy

Negative-ion electrospray ionization mass spectrometry (ESI-MS) and ESI-MS/MS analysis were performed on a Micromass LTQ-Orbitrap XL instrument (Thermo Fisher Scientific, Waltham, Massachusetts, MA, USA). Ten microliters of sample dissolved in acetonitrile/H2O (1:1, *v*/*v*) was injected, and the mobile phase (acetonitrile/H_2_O, 1:1, *v*/*v*) was delivered by a syringe pump at a flow rate of 10 μL/min [24].

### 2.6. NMR Spectroscopy Analysis

The ^1^H NMR spectra of HAOs were recorded at 25 °C on an Agilent DD2-500 spectrometer (500 MHz, Santa Clara, CA, USA). All samples were previously dissolved in deuterium oxide (D_2_O, 99.96%) and lyophilized three times to replace exchangeable protons. The lyophilized samples were then dissolved in D_2_O at a concentration of 20 g/L.

### 2.7. 1-Phenyl-3-methyl-5-pyrazolone (PMP) Labeling

A sample of HAOs (5 mg) was dissolved in 0.3 mol/L NaOH aqueous solution (200 μL). Then, PMP methanol solution (240 µL, 0.5 mol/L) was added for PMP labeling. The labeling reaction was conducted at 70 °C for 1 h and then cooled to room temperature followed by neutralization using acetic acid. The extra PMP in the labeling reaction was extracted by using 1.0 mL chloroform three times, as previously described [25].

### 2.8. Synthesis of Hyaluronic Acid Conjugated with Fluorescein Isothiocyanate (FITC) (HA-FITC) 

HA (400 mg) was dissolved in 20 mL hexamethylene diamine (0.5 mol/L) in 10% acetic acid solution. Then, sodium borohydride aqueous solution (150 mg/mL, 2 mL) was added to the HA solution. After stirring at 80 °C for 12 h, the supernatant was adjusted to pH 8.5 by Na_2_CO_3_ dilute solution. And then the FITC methanol solution (15 mg/mL) was added dropwise. After stirring for 24 h at 37 °C, HA-FITC was obtained after diafiltration by using a 1 kDa cut-off dialysis membrane and freeze-drying.

### 2.9. Binding Assay of HA Oligosaccharides (HAOs) to MDA-MB-231 Cells by Flow Cytometry Analysis

Flow cytometry analysis was performed as described previously [26]. The MDA-MB-231 cell binding assay was performed using HA-FITC as competing probe. About 2 × 10^5^ cells per well were seeded in a 12-well plate. The cells were preincubated with HAOs (100 mmol/L) for 1 h at room temperature with constant shaking. HA-FITC (2 mg/mL) was added to the mixture for 1 h under constant shaking in the dark. Cells incubated with unlabeled HA served as a negative control, whereas cells incubated with HA-FITC alone served as a positive control. After incubation, the supernatant was removed by centrifugation. A total of 1 × 10^4^ gated events were acquired per sample, and the mean fluorescence intensity was plotted in a histogram-based graphical representation. Data were analyzed with FlowJo_V10 software (10.5.4, Tree Star, San Carlos, Chicago, CA, USA).

### 2.10. Molecular Docking of HAOs

Molecular docking was performed by using Molecular Operating Environment (MOE) software (2015.10, Chemical Computing Group, Montreal, Canada). The X-ray diffraction crystal structures of mouse CD44 (ID: 2JCQ) and mouse TLR4 (ID: 2Z64) were obtained from the Protein Data Bank. There is similarity in binding of HA between mouse and human active sites [27,28,29]. Three-dimensional structures of HAOs were generated by MOE. The protein structures were prepared using the QuickPrep module of MOE, and the energies were minimized through the General method at a 0.1 kcal mol^−1^A˚^−2^ Root Mean Square (RMS) Gradient. The docking poses were scored using a London △G scoring function with five parameters including rotational and translational entropy, ligand flexibility, hydrogen bonding, metal ligations, and desolvation energy. At least 30 poses for each compound were retained and ranked via the Generalized-Born Volume Integral/Weighted Surface area (GBVI/WSA dG) (Chemical Computing Group, Montreal, QC, Canada) scoring function [30].

### 2.11. Viability Assay of MDA-MB-231 Cells

MDA-MB-231 cells were incubated in 96-well polystyrene cell culture plates for 24 h. The cells were incubated with HAOs (100 mmol/L) for 48 h at 37 °C. Afterwards, the cells were incubated for 16 h with 2 mg/mL resazurin solution at 37 °C, and then the fluorescence intensity was measured using a SpectraMax M3 microplate reader (Molecular Devices, San Jose, CA, USA) at an excitation wavelength of 544 nm and emission wavelength of 595 nm.

### 2.12. Migration Assay of MDA-MB-231 Cells

The migration of MDA-MB-231 cells was investigated as described previously with modifications [31]. Viable cells were plated at 5 × 10^4^ cells per well in 24-well culture plates using growth media containing 10% FBS. After the cells had attached, scratch wounds were created by scraping the cell monolayers with a 1000 µL sterile pipette tip. The cells were treated with different HAOs in DMEM after washing away suspended cells. An equal volume PBS aqueous solution was added as control wells. Photomicrographs were taken at 48 h with a ZEISS Vert.A1 microscope (Carl Zeiss, Heidenheim, Germany) equipped with a digital camera. Tracings were digitized by scanning, and the wound areas were quantified using Image J software (v1.8.0, National Institutes of Health, Bethesda, Maryland, ME, USA). 

### 2.13. Statistical Analysis 

All of the experimental data were performed in triplicate and expressed as means ± SD, and statistical significance was calculated using GraphPad Prism 5.0 software (GraphPad Software Inc., San Diego, CA, USA). Comparisons between groups were performed using one-way ANOVA analysis followed by Tukey’s tests while considering a *p* value < 0.05 as statistically significant.

## 3. Results 

### 3.1. Preparation of HA Oligosaccharides by Acid Hydrolysis

**Figure 1:**
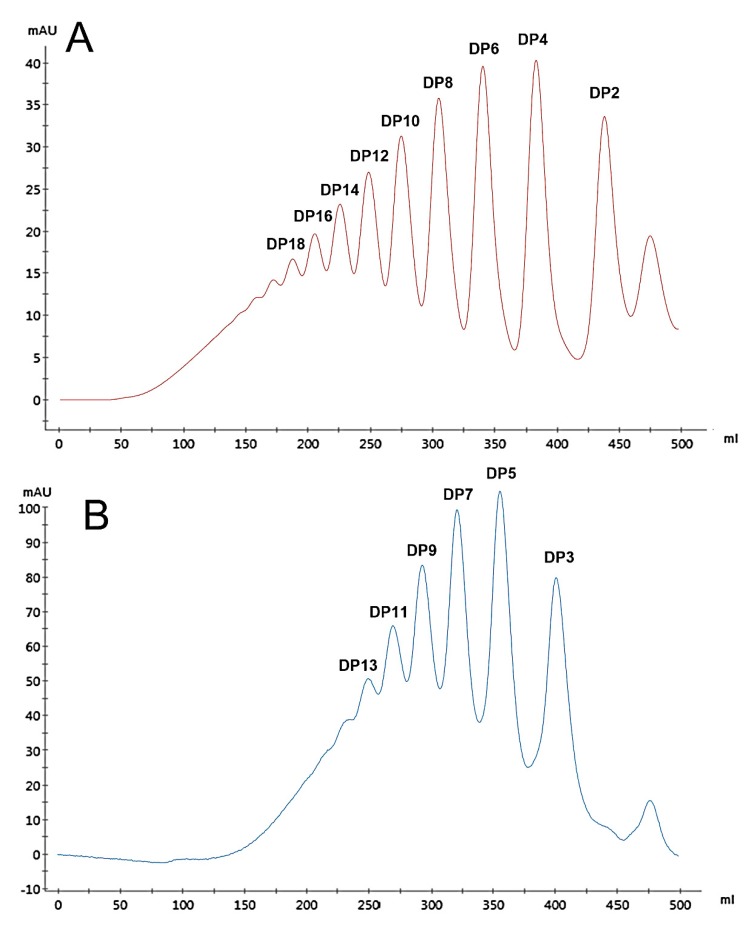
Hyaluronic acid oligosaccharide 1 (HAO1) (**A**) and HAO2 (**B**) were fractionated by gel exclusion chromatography on a Bio-Gel P10 column.

Two series of HAOs were prepared by 1.0 and 0.1 mol/L hydrochloric acid hydrolysis, respectively, followed by separation using gel exclusion chromatography (Figure 1A,B). The data in Figure 1 show that HAO1 did not contain the fractions of HAO2, and HAO2 did not contain the fractions of HAO1. After further purification of each HAO fraction, the purity of all HAOs exceeded 95.0% (as shown in Table 1), which were analyzed by HPLC and calculated by the peak area ratio.

### 3.2. The Negative-Ion Negative-Ion Electrospray Ionization Mass Spectrometry (ESI-MS) of HA Oligosaccharide Fractions 

**Figure 2:**
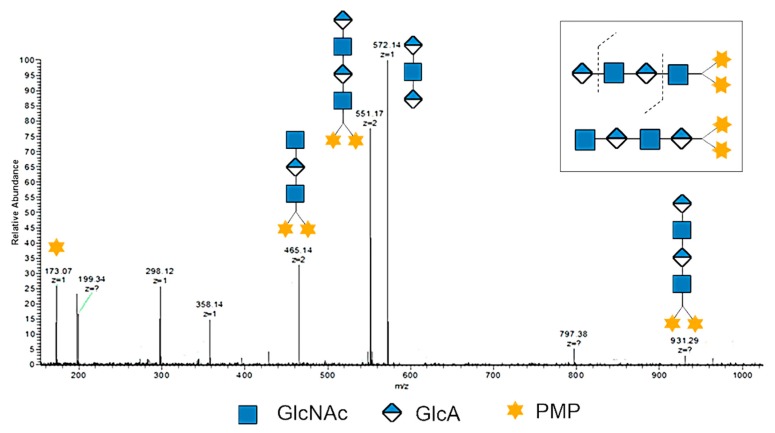
The negative-ion ESI-MS/MS profile of DP4 with labeled 1-phenyl-3-methyl-5-pyrazolone (PMP). The ion of *m*/*z* 551.17 was selected as the precursor for negative-ion ESI-MS/MS analysis.

Both HAO1 and HAO2 were analyzed by negative-ion ESI-MS. The data (as shown in Table 1, Figures S1–S10) suggest that HAO1s are the even-numbered oligosaccharides (DP2-10) with GlcNAc at the reducing end, while HAO2s are the odd-numbered oligosaccharides (DP3-11) with glucuronic acid at the reducing end. Taking the odd-numbered DP3 as an example, there were two types of possible DP3 sequences in theory, GlcA-GlcNAc-GlcA (molecular mass: 573.15) and GlcNAc-GlcA-GlcNAc (molecular mass: 776.23). Based on the ESI-MS data (Table 1), the ions of [M – 2H]^2−^ (*m*/*z* 285.58) and [M − H]^−^ (*m/z* 572.14) suggest the presence of a GlcA-GlcNAc-GlcA structural unit and no glycosidic fragment ions of GlcNAc-GlcA-GlcNAc. However, with the even-numbered HAOs, two isomers may exist in the ESI-MS analysis. To make the structure of even-numbered HAOs clear, we applied a PMP labeling strategy, which only labeled at the C1 of the reducing end to distinguish the isomers. Taking the PMP labeled even-numbered DP4-2PMP as an example, the doubly charged ion [M – 2H]^2−^ (*m/z* 551.17) of HAO was selected as the precursor for negative-ion ESI-MS/MS analysis to deduce its sequence. As shown in Figure 2, a set of glycosidic fragment ions of PMP at *m*/*z* 173.07, [M – GlcA − 2H]^2−^ at *m*/*z* 465.14, [M −2H]^2−^ at *m/z* 551.17, [M − 2PMP − GlcNAc − 2H]^−^ at *m*/*z* 572.14, and [M −H]^−^ at *m/z* 931.29 suggest the sequence structure of GlcA-GlcNAc-GlcA-GlcNAc. More importantly, no glycosidic fragment ions of the GlcNAc-GlcA-GlcNAc-GlcA sequence were observed. Therefore, all the even-numbered HAOs were confirmed to have a structure of GlcA at the nonreducing end and the GlcNAc residue at the reducing end. 

### 3.3. NMR Spectroscopy of HA Oligosaccharide Fractions

**Figure 3:**
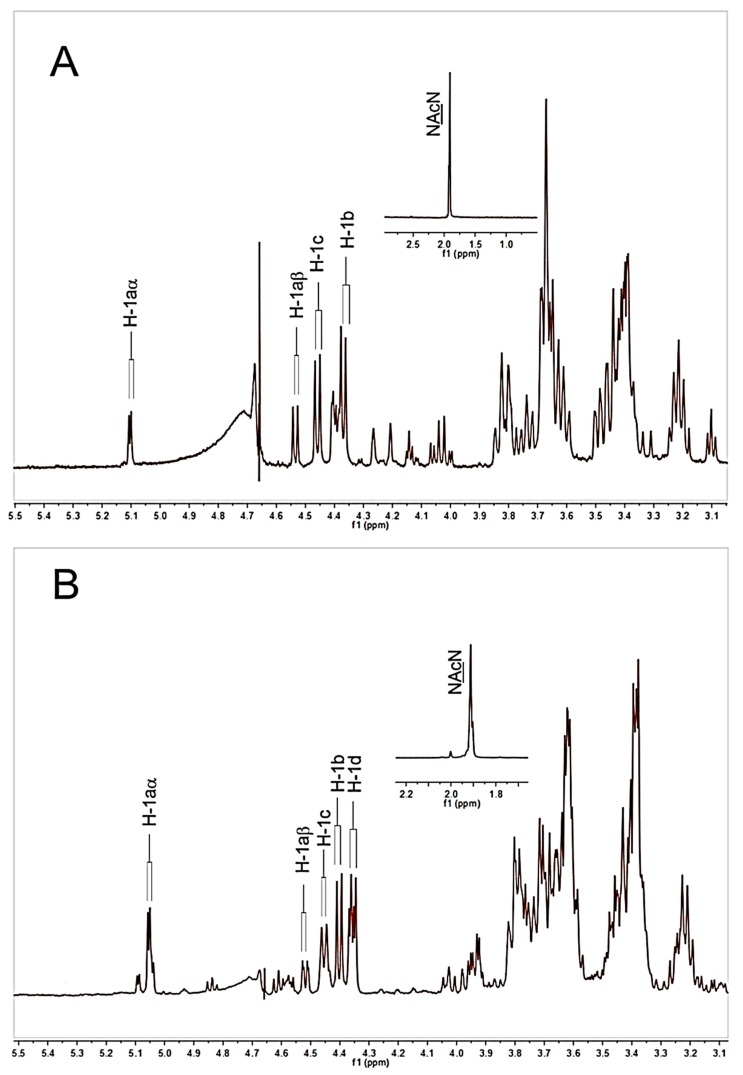
^1^H NMR spectra of HAOs. (**A**) DP3, (**B**) DP4. All samples were previously dissolved in deuterium oxide (D_2_O, 99.96%) and lyophilized three times to replace exchangeable protons.

The ^1^H-NMR spectra of HAOs were used to characterize the odd- and even-numbered oligosaccharide fractions (Figures S11–S20). The ^1^H-NMR data assignments of DP3 and DP4 were taken as examples. Four anomeric signals of DP3 (Figure 3A) were observed at δ5.11, 4.54, 4.47, and 4.40 ppm, which can be attributed to H-1aα (reducing end GlcA), H-1aβ (reducing end GlcA), H-1c (interior GlcNAc), and H-1b (nonreducing terminal GlcA), respectively, as previously described [32]. The signal at 1.92 ppm corresponds to the N-acetyl signal of the interior GlcNAc of DP3. These data are in accordance with the sequence of GlcA-GlcNAc-GlcA in DP3. Similarly, the anomeric signals of DP4 are assigned as shown in Figure 3B, which are also in accordance with the sequence of GlcA-GlcNAc-GlcA-GlcNAc in DP4.

### 3.4. Glycosidic Bond-Selective Depolymerization by Acid Hydrolysis

**Figure 4:**
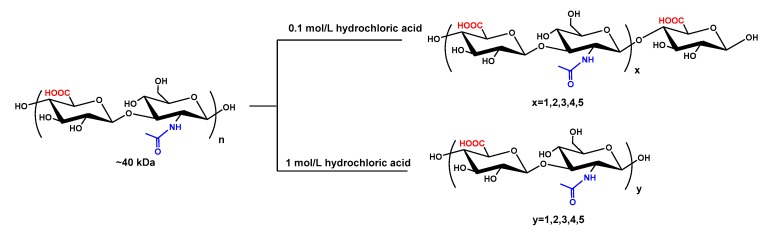
Preparation of HAOs. The purified HA (~40 kDa) was cleaved with hydrochloric acid at concentrations of 0.1 and 1 mol/L and then fractionated by GPC to obtain the even- and odd-numbered oligosaccharides, respectively.

Interestingly, based on both ESI-MS and NMR analyses of HAOs, the odd-numbered and even-numbered HAOs were obtained for the first time by hydrochloric acid hydrolysis at the concentrations of 0.1 and 1 mol/L, respectively (as shown in Figure 4). The development of a glycosidic bond-selective depolymerization method is critical to obtain HAOs with specific DP. The glycosidic bond-selective oligosaccharides were prepared by a chemical approach and enzymatic process [33,34]. Acid hydrolysis has been studied as a way for producing oligosaccharides randomly by Tokita [35]. However, two series of HAOs with different DP and reducing ends were selectively produced in our experiments only by changing the hydrochloric acid concentration. We deduced that the mechanism may be the difference in the stability between the β (1→4) glycosidic bond and the β (1→3) glycosidic bond under acidic conditions. The β (1→3) glycosidic bond is liable to cleave at a low concentration of hydrochloric acid, and then the odd-numbered HAOs were selectively obtained by 0.1 mol/L hydrochloric acid hydrolysis. While the β (1→4) glycosidic bond was mainly cleaved at high concentration of hydrochloric acid, the even-numbered HAOs were selectively obtained by 1.0 mol/L hydrochloric acid hydrolysis. 

### 3.5. Binding Abilities of HAOs with MDA-MB-231 Cells

**Figure 5:**
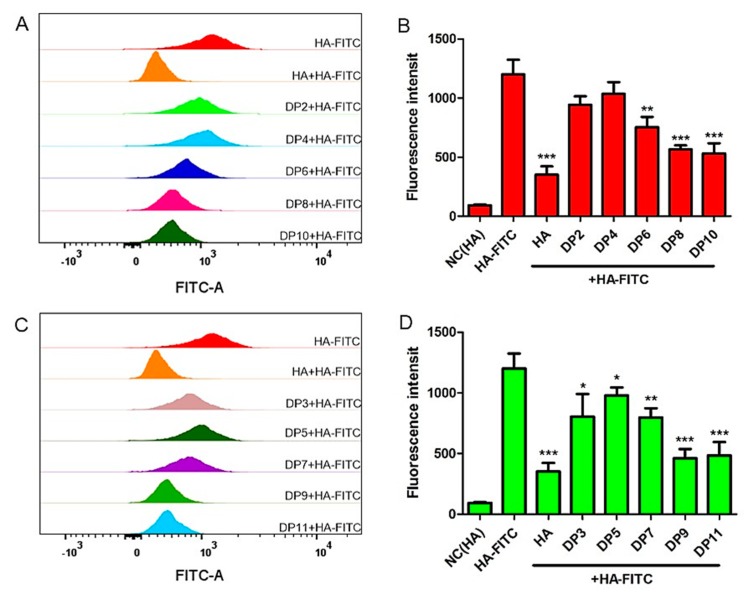
The binding abilities between HAOs and MDA-MB-231. (**A**) Flow-cytometry histograms showing the ability for the even-numbered HAOs to block HA-fluorescein isothiocyanate (FITC) binding of MDA-MB-231 cells. (**B**) Fluorescence intensities obtained after competitive binding of even-numbered HAOs during flow-cytometry analysis. (**C**) The binding abilities of the odd-numbered HAOs. (**D**) Fluorescence intensities of the odd-numbered HAOs. Data represents the mean ± SD with reference to the HA-FITC group, * *p* < 0.5, ** *p* < 0.01, *** *p*< 0.001; one-way ANOVA with Tukey’s multiple comparisons test.

Based on the larger amount of HA that was observed to accumulate in extracellular fluid of TNBC, we deduced that HAOs may influence TNBC growth by binding with the cell surface receptors. To investigate the binding abilities of HAOs with MDA-MB-231 TNBC cells, we designed a competitive binding assay where the HAOs were initially preincubated with the cells for 1 h, followed by incubation with HA-FITC for another 1 h, to allow the binding competition of HAOs with the native ligand. As shown in Figure 5, a higher fluorescence intensity was observed in each HAO than that of the cells incubated with HA, indicating that HAOs can bind with MDA-MB-231 TNBC cell surface receptors. In addition, the binding fluorescence intensity was observed to increase with the increase in DP of the HAOs. 

### 3.6. Molecular Docking of HAOs

**Figure 6:**
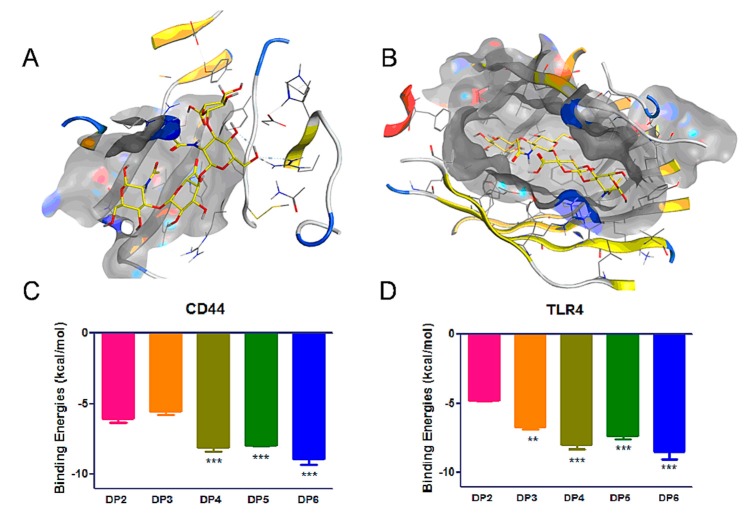
Molecular docking by MOE software of HAOs with receptors. Reasonable binding poses of HA-DP4 in CD44 (**A**) and TLR4 (**B**) pockets. Binding energies of HAOs with CD44 (**C**) and TLR4 (**D**) pockets. Data represent the mean ± SD with reference to the DP2 group, ** *p* < 0.01, *** *p* < 0.001; one-way ANOVA with Tukey’s multiple comparisons test.

The CD44 and TLR4 receptors play important roles in MDA-MB-231 biological functions, such as migration, adhesion, and proliferation processes. To probe the binding ability of HAOs with these receptors, we used the dock module of MOE software to perform molecular docking simulations of HAOs with CD44 and TLR4. Due to the software design for small molecular compounds, we were able to provide the data for small molecular HA (DP2-DP6). The binding poses of DP4 in the binding pocket are displayed in Figure 6A,B and the binding energies of DP2-DP6 are displayed in Figure 6C,D. These data showed that the binding energies of HAOs with CD44 and TLR4 increased as the DP of HAOs increased, indicating that the DP was a key determining factor for the HA to bind with receptors. Interestingly, the binding energies significantly increased when a GlcNAc residue was added to the reducing end of HAOs, for example, the DP4 showed a higher binding energy (–8.1 kcal mol^−1^) than the binding energy of DP3 (–5.6 kcal mol^−1^) in the CD44 pocket. However, the binding energies showed no remarkable changes when a GlcA residue was added to the reducing end of HAOs, for example, the DP4 and DP5 showed similar binding energies with the CD44 receptor (–8.1 and –7.9 kcal mol^−1^, respectively). These data suggest that the GlcNAc residue plays an important role in the binding of HAOs with CD44. In addition, our results are also in accordance with the reports that the binding energies will reduce in deacetylated HA [26,36]. The interactions between HAOs and its receptors predicted that HAOs might affect the migration and growth of MDA-MB-231 cells. 

### 3.7. Effect of HAOs on MDA-MB-231 Migration and Growth

**Figure 7:**
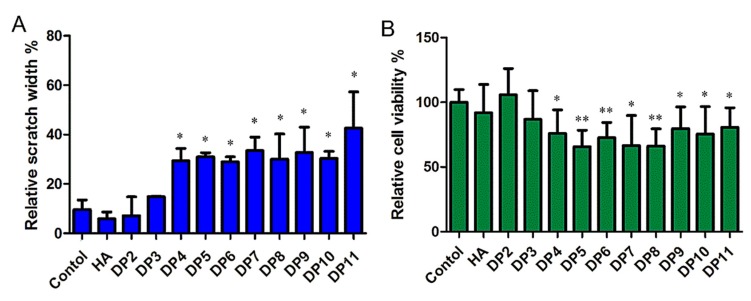
The migration and growth of MDA-MB-231 induced by HAOs. (**A**) Scratch width of the control in the presence of HAOs at 100 mmol/L for 48 h. (**B**) Relative cell viability after 48 h treatment with 100 mmol/L HAOs. Data represent the mean ± SD with reference to the PBS treatment group. * *p* < 0.05, ** *p* < 0.01; one-way ANOVA with Tukey’s multiple comparisons test.

We tested the effect of HAOs on MDA-MB-231 migration and growth in vitro by a scratch assay (as shown in Figure 7A), and significant differences were observed in differently sized HAOs. DP2 and DP3 showed negligible effects on MDA-MB-231 cell migration. However, significant inhibitions of MDA-MB-231 cell migration were observed from DP4 to DP11. In addition, we also evaluated the effect of HAOs on MDA-MB-231 cell growth (as shown in Figure 7B) by a resazurin test, and we found that both the even- and odd-numbered HAOs (except DP2 and DP3) significantly inhibited MDA-MB-231 cell growth.

## 4. Discussion

The size of HA plays a crucial role in the modulation of different biological processes because it affects interactions with cell receptors such as CD44, RHAMM, and TLRs [37,38,39]. Therefore, various methods were reported to obtain HAOs with defined sizes and structures such as physical methods, chemical approaches, and enzymatic processes [40,41,42]. In our research, a series of HAOs were obtained by glycosidic bond-selective depolymerization. HAOs with specific DP that were well-characterized were applied to evaluate the biological activity.

Our results have shown that the biological function related to the size of HA fragments. Moreover, the inhibition of MDA-MB-231 grew nonlinearly as the size of the HAOs increased (Figure 7). The data in Figure 5 show that all HAOs interacted with MDA-MB-231 cells. The DP2 and DP5 possessed weaker binding abilities than longer HAOs. More importantly, the molecular docking data in Figure 6 suggest that the binding energies of HAOs with receptors are closely related to DP, which is in accordance with the results of HAO biological functions. The HA tetrasaccharide (HAO-DP4) was confirmed to be the minimum fragment necessary to inhibit TNBC. These similar results were also demonstrated in other GAG oligosaccharides, for example, tetra oligosaccharide is the shortest heparan sulfate that binds to fibroblast growth factor 2 (FGF2) [43]. DP2 and DP3 may be too small to activate the receptors’ functions. The structure and order of sugar residues also play important roles for GAG biological functions [6,44]. Our results suggest that the order of GlcA or GlcNAc was not the essential effect on the MDA-MB-231 of cell growth. However, the difference may appear in other activities such as inflammation, wound healing, tissue injury, and fibroblasts.

Our results showed that HAOs inhibited tumor growth, which was in agreement with Zeng and collaborators [21]. On the contrary, the “low molecular HA (LMW-HA)” was reported to be pro-oncogenic [45]. This difference may be relating to the size of HA, for HAOs in our experiments had lower molecular masses than those of “LMW-HA”. HAOs normally appear in the wound area and are degraded by hydroxyl radicals or bacterial enzymes in the human body [46]. Moreover, recent reports show that small and large carbohydrate ligands have different binding sites for langerin receptors [47]. HAOs and HA may bind different sites to receptors and show different functions. However, further studies are needed to confirm these hypotheses.

## 5. Conclusions

In summary, both odd- and even-numbered HAOs were obtained by bond-selective degradation for the first time. A total of 10 different HAOs (DP2-10 and DP3-11) with high purity were fractionated by GPC. Their structures were characterized by means of HPLC, ESI-MS, and NMR. Biological evaluation indicated that all HAOs significantly inhibited the growth and migration of TNBC MDA-MB-231 cells. Among these oligosaccharides, DP4 was confirmed to be the minimum fragment necessary to inhibit MDA-MB-231 cells. We would like to further study whether an in vitro situation can also be applied to in vivo conditions. Our data provide a basis for further research on the structural activity relationship of HAOs.

## Figures and Tables

**Table 1:** Summary of the results of ESI-MS and HPLC measurements of HAOs.

Fraction	Rf ^a^	[M −H]^−^	[M −2H]^2−^	[M −3H^]3−^	[M −4H]^4−^	Molecular Mass	Purity (%) ^b^
DP2	0.79	396.20				397.33	99.5
DP3	0.76	572.17	285.58			573.46	98.4
DP4	0.54	775.22	387.11			776.65	99.3
DP5	0.53		475.15	316.43		952.78	98.6
DP6	0.36		576.66	384.11		1155.79	99.5
DP7	0.32		664.71	442.81		1332.09	98.0
DP8	0.23		766.60			1535.29	99.0
DP9	0.18		854.28	569.18	426.64	1711.41	99.4
DP10	0.15		956.20	637.20		1914.61	98.8
DP11	0.11			695.56	521.42	2090.73	98.1

^a^ Retardation factor (Rf) determined by thin layer chromatography. ^b^ Oligosaccharide purity determined by HPLC.

## Supplementary Materials

The following are available online at https://www.mdpi.com/2218-273X/9/9/436/s1, Figure S1: The negative-ion ESI-MS of 3-mer HA, Figure S2: The negative-ion ESI-MS of 5-mer HA, Figure S3: The negative-ion ESI-MS of 7-mer HA, Figure S4: The negative-ion ESI-MS of 9-mer HA, Figure S5: The negative-ion ESI-MS of 11-mer HA, Figure S6: The negative-ion ESI-MS of 2-mer HA, Figure S7: The negative-ion ESI-MS of 4-mer HA, Figure S8: The negative-ion ESI-MS of 6-mer HA, Figure S9: The negative-ion ESI-MS of 8-mer HA, Figure S10: The negative-ion ESI-MS of 10-mer HA, Figure S11: The 1H NMR spectrum of 3-mer HA, Figure S12: The 1H NMR spectrum of 5-mer HA, Figure S13: The 1H NMR spectrum of 7-mer HA, Figure S14: The 1H NMR spectrum of 9-mer HA, Figure S15: The 1H NMR spectrum of 11-mer HA, Figure S16: The 1H NMR spectrum of 2-mer HA, Figure S17: The 1H NMR spectrum of 4-mer HA, Figure S18: The 1H NMR spectrum of 6-mer HA, Figure S19: The 1H NMR spectrum of 8-mer HA, Figure S20: The 1H NMR spectrum of 10-mer HA.
