# Supplementary material for: Preparation, Characterization, and Inhibition of Hyaluronic Acid Oligosaccharides in Triple-Negative Breast Cancer
Source: Biomolecules. 2019 Sep 1;9(9):436. doi: 10.3390/biom9090436 (PMC6770828; doi:10.3390/biom9090436)
Supplement: Supplementary file 1 [file biomolecules-09-00436-s001.pdf]

Supplementary information

1.The Negative-Ion ESI-MS of HAOs with Even-Numbered Sugar Residues

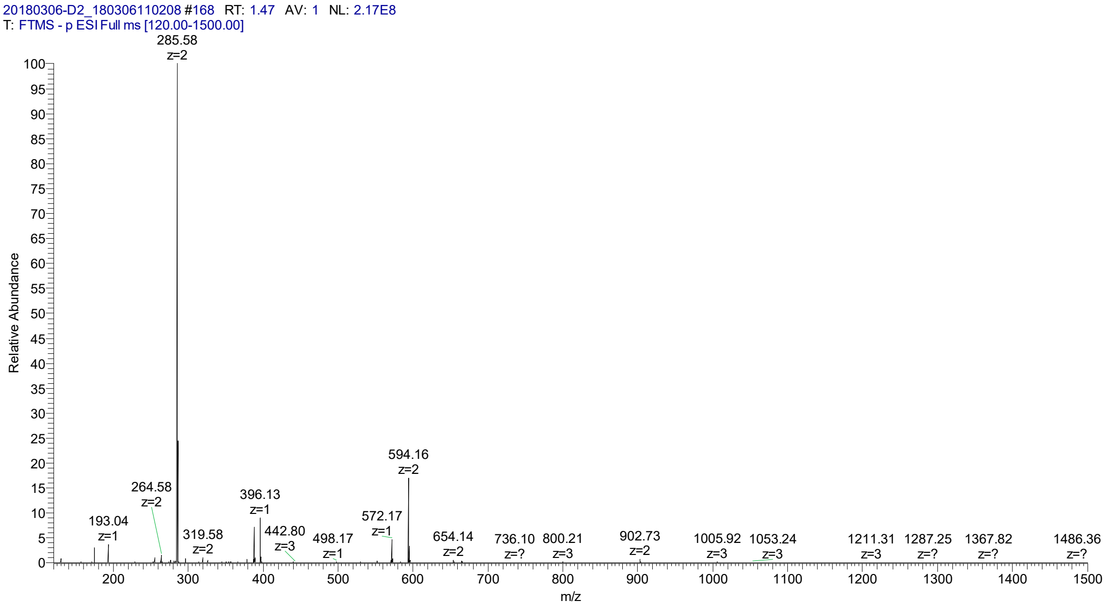

Figure S1. The negative-ion ESI-MS of 3-mer HA

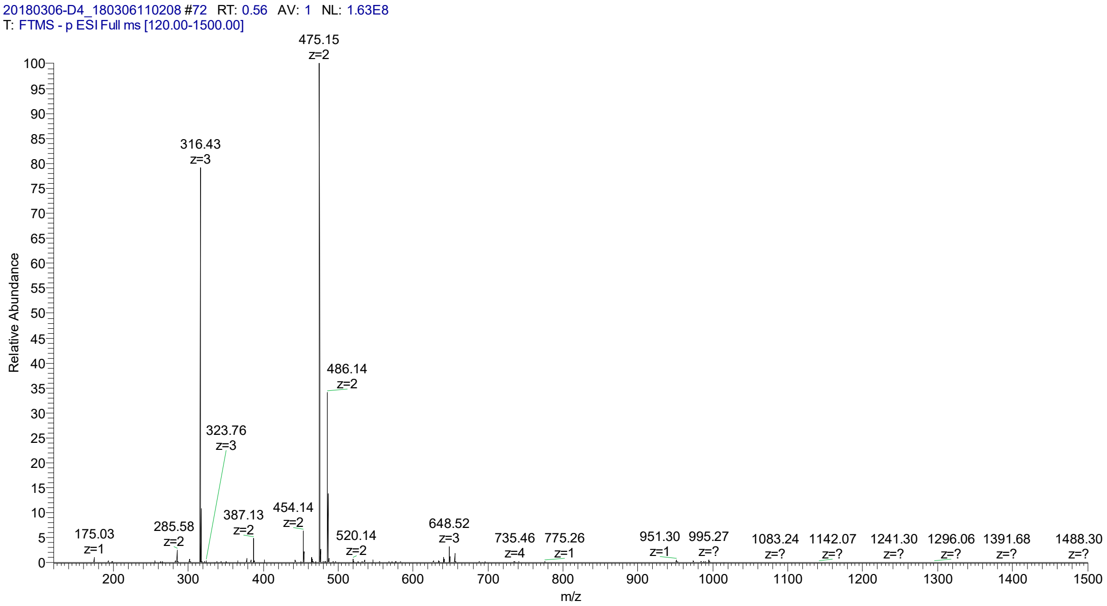

Figure S2 The negative-ion ESI-MS of 5-mer HA

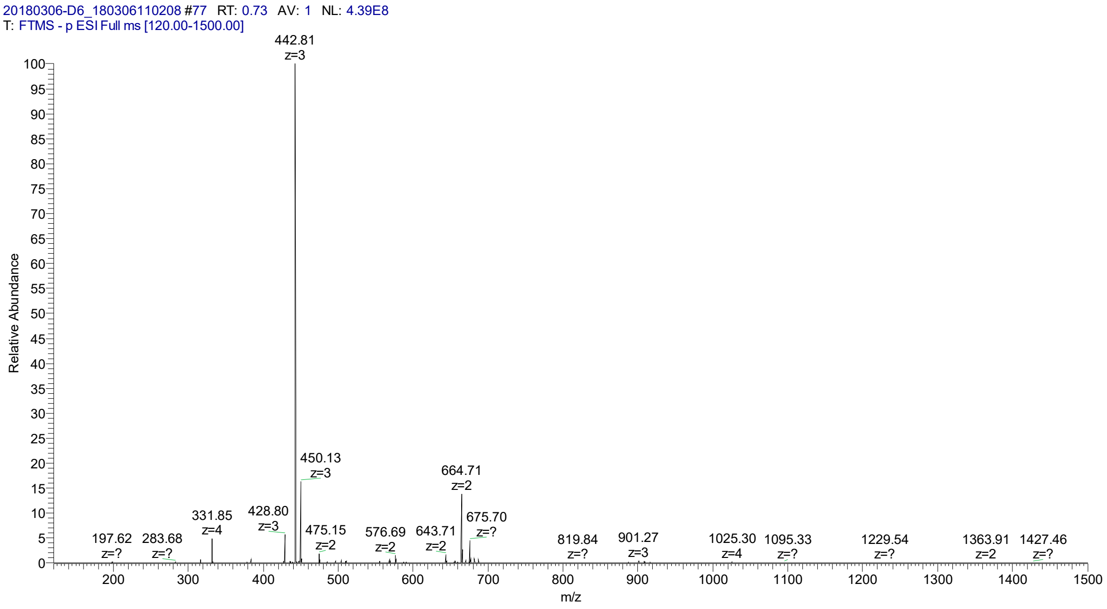

Figure S3. The negative-ion ESI-MS of 7-mer HA

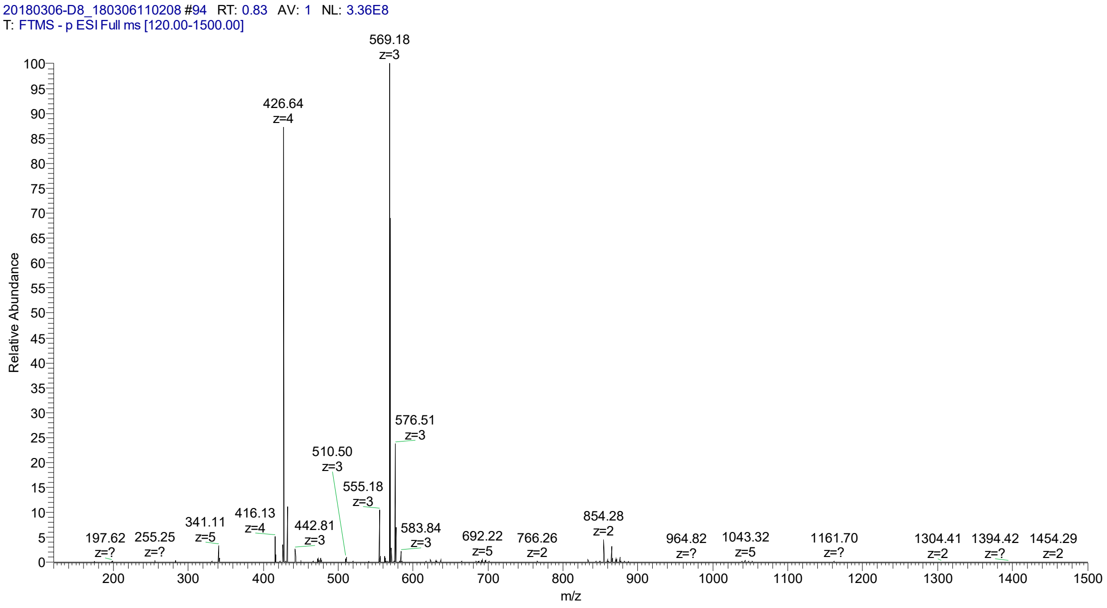

Figure S4. The negative-ion ESI-MS of 9-mer HA

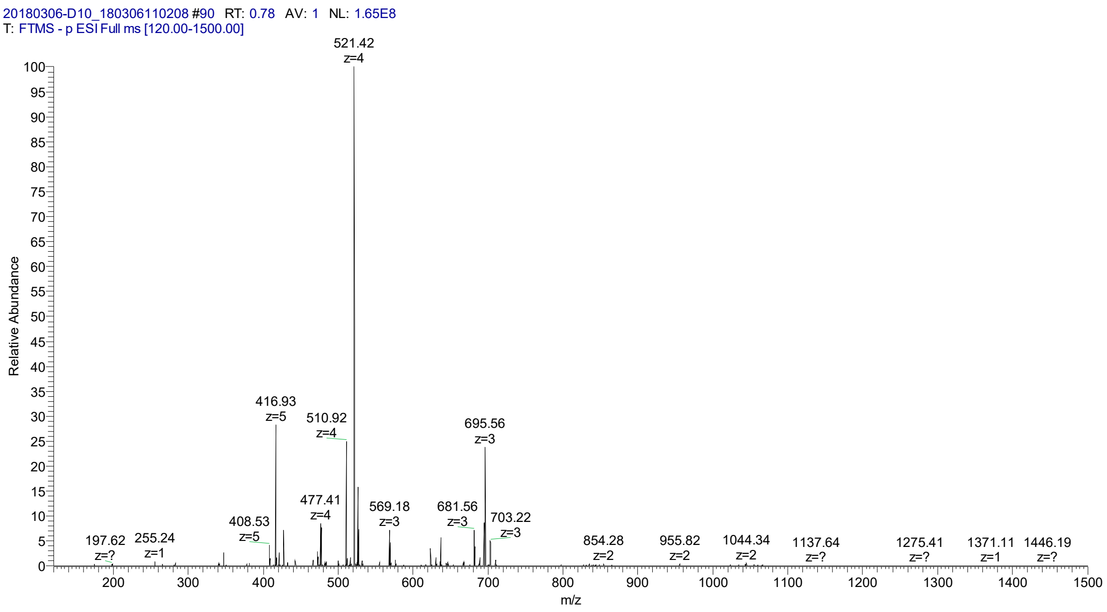

Figure S5. The negative-ion ESI-MS of 11-mer HA

2.The negative-ion ESI-MS of HAOs with odd-numbered sugar residues

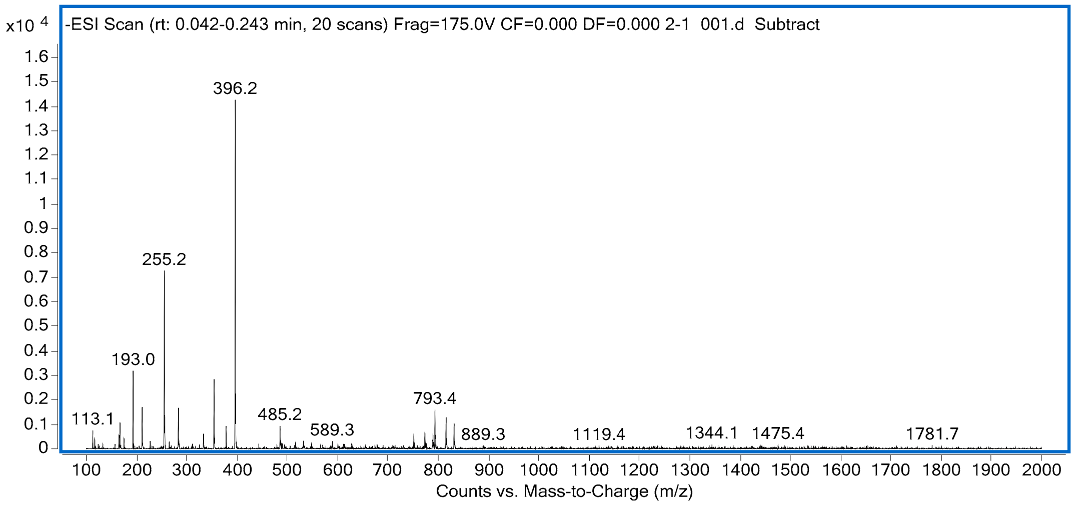

Figure S6. The negative-ion ESI-MS of 2-mer HA

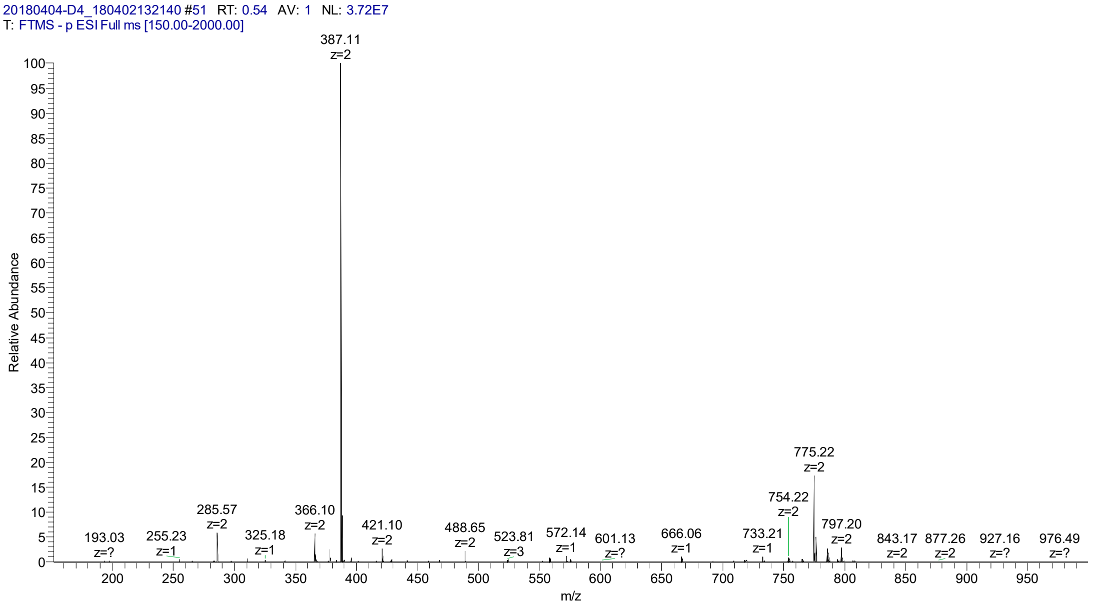

Figure S7. The negative-ion ESI-MS of 4-mer HA

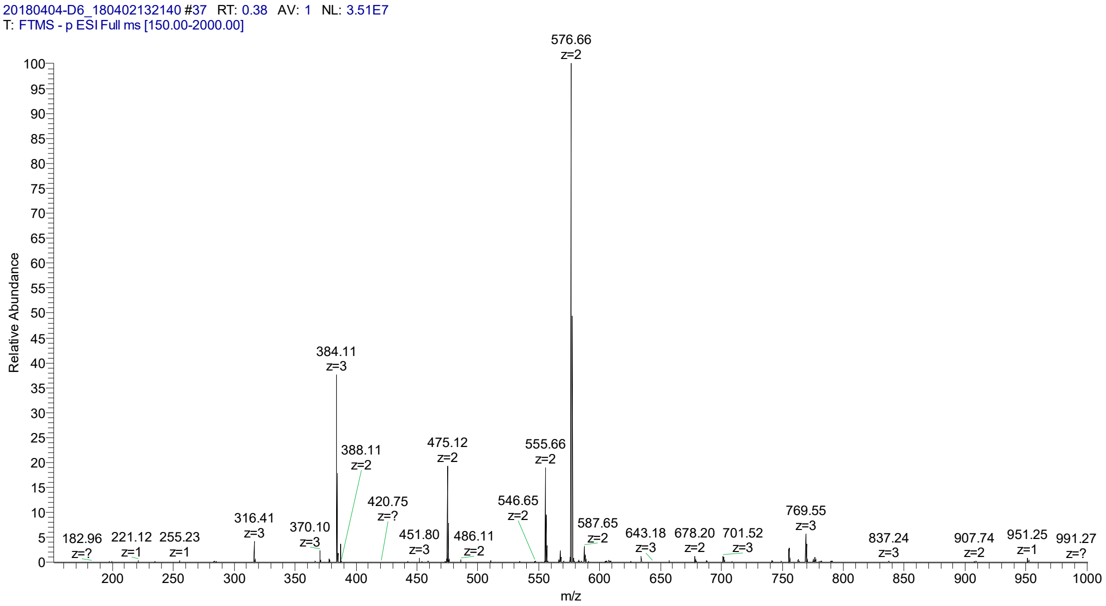

Figure S8. The negative-ion ESI-MS of 6-mer HA

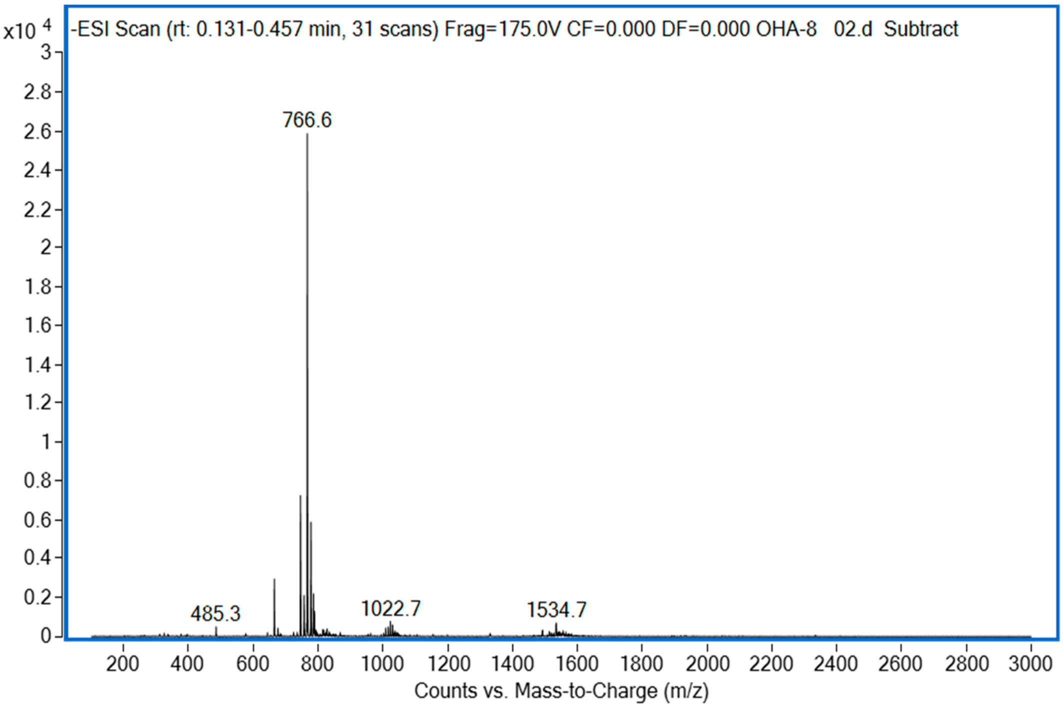

Figure S9. The negative-ion ESI-MS of 8-mer HA

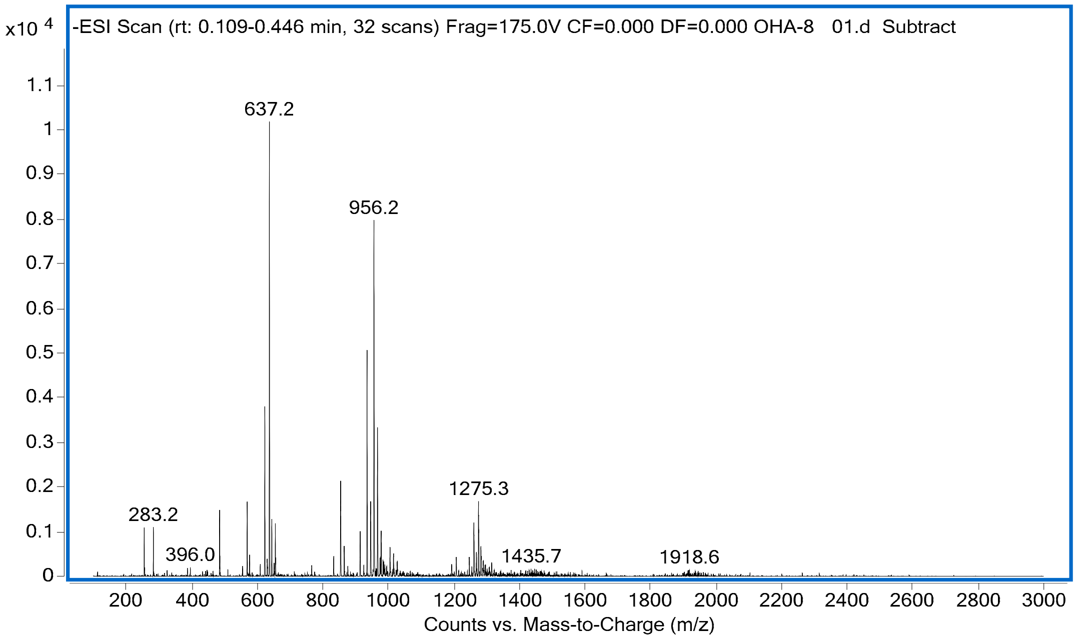

Figure S10. The negative-ion ESI-MS of 10-mer HA

3.The ^{1}H NMR spectra of HAOs with even-numbered sugar residues

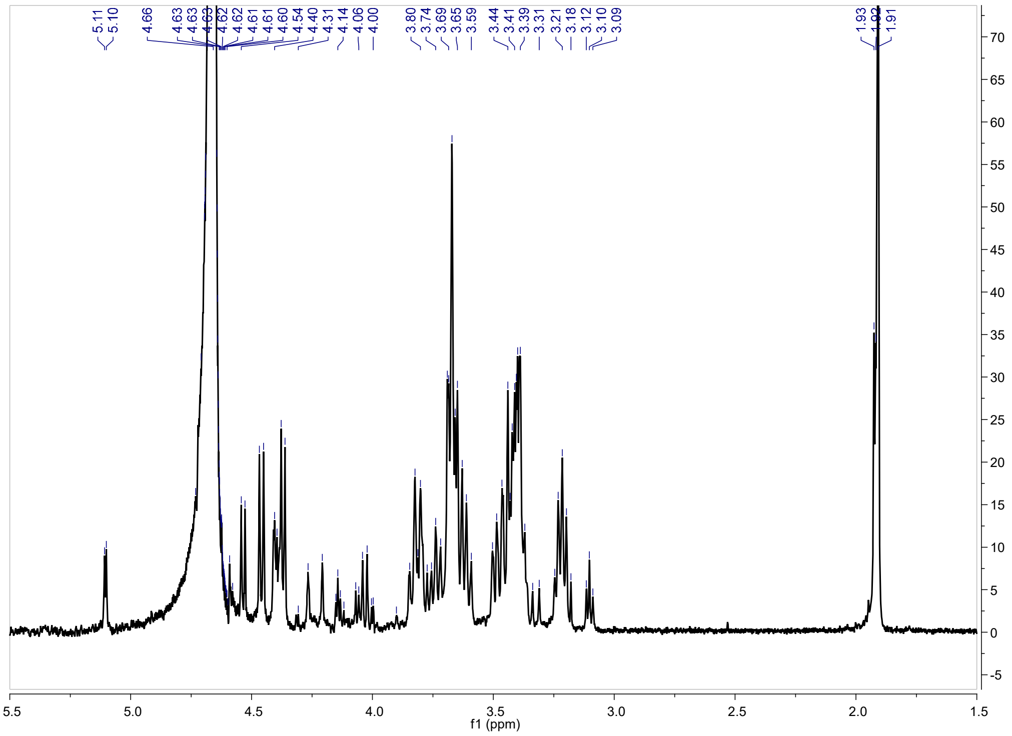

Figure S11. The ¹H NMR spectrum of 3-mer HA

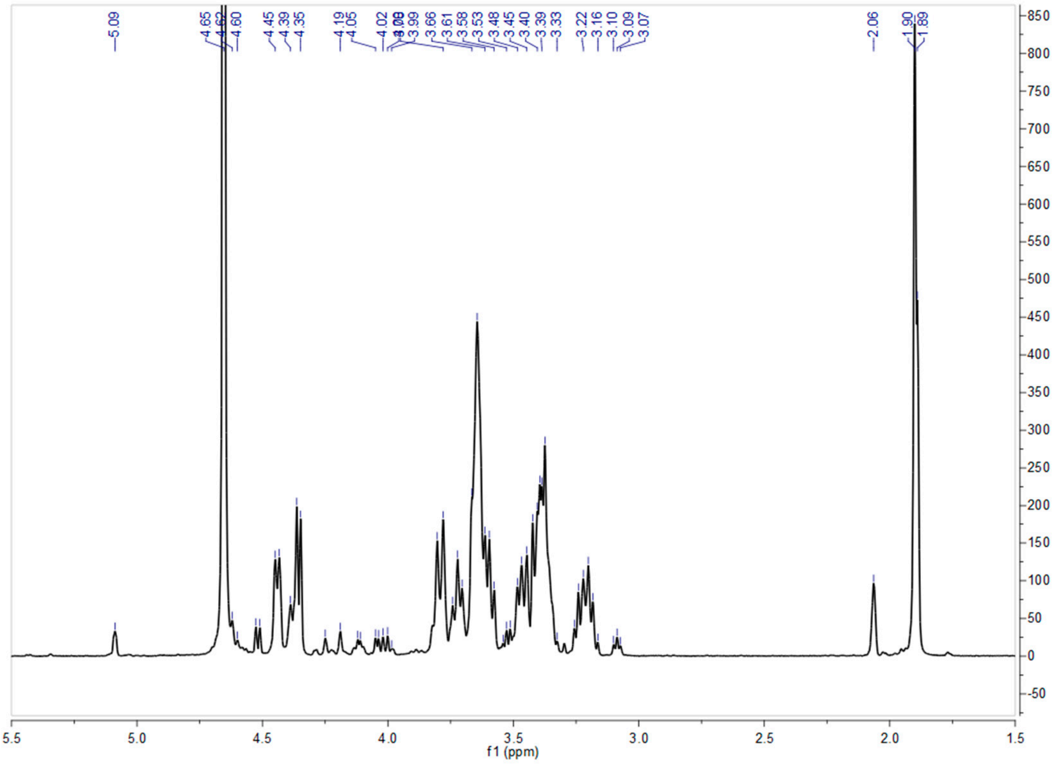

Figure S12. The ¹H NMR spectrum of 5-mer HA

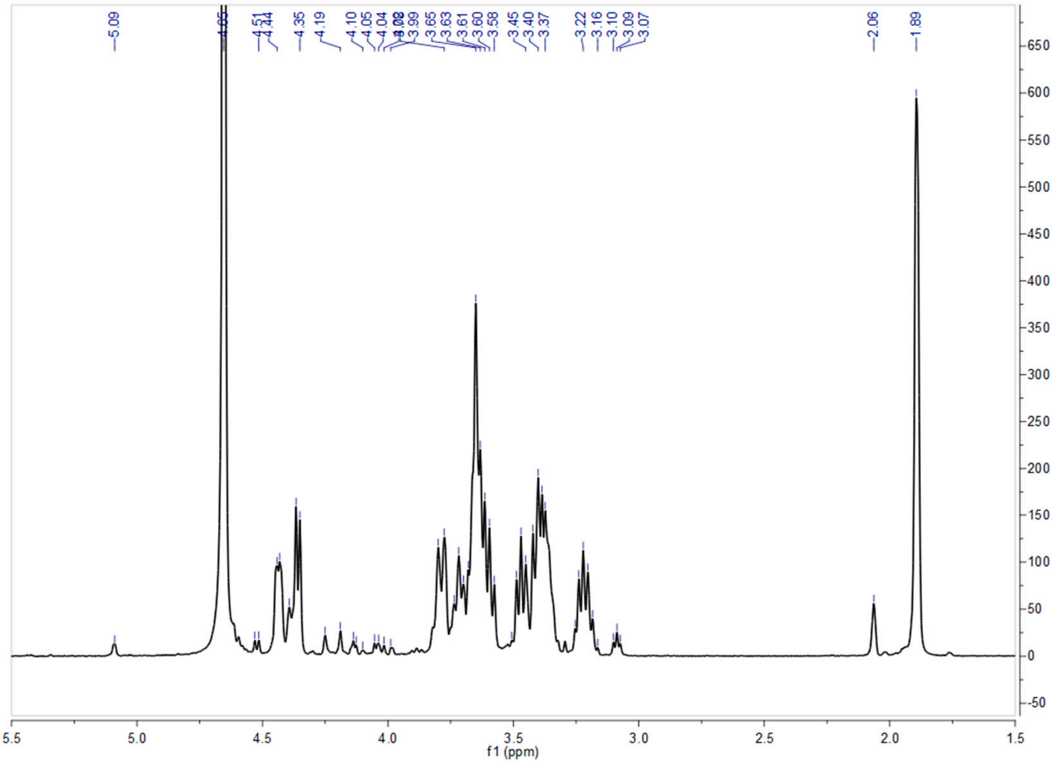

Figure S13. The ^{1}H NMR spectrum of 7-mer HA

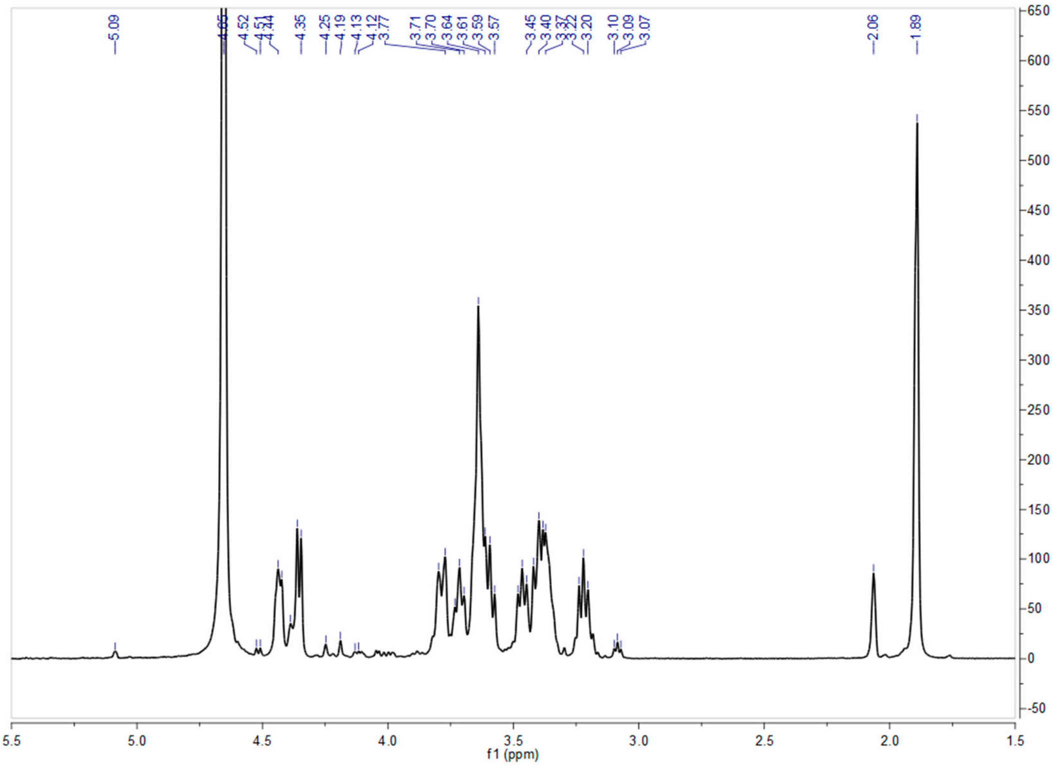

Figure S14. The ^{1}H NMR spectrum of 9-mer HA

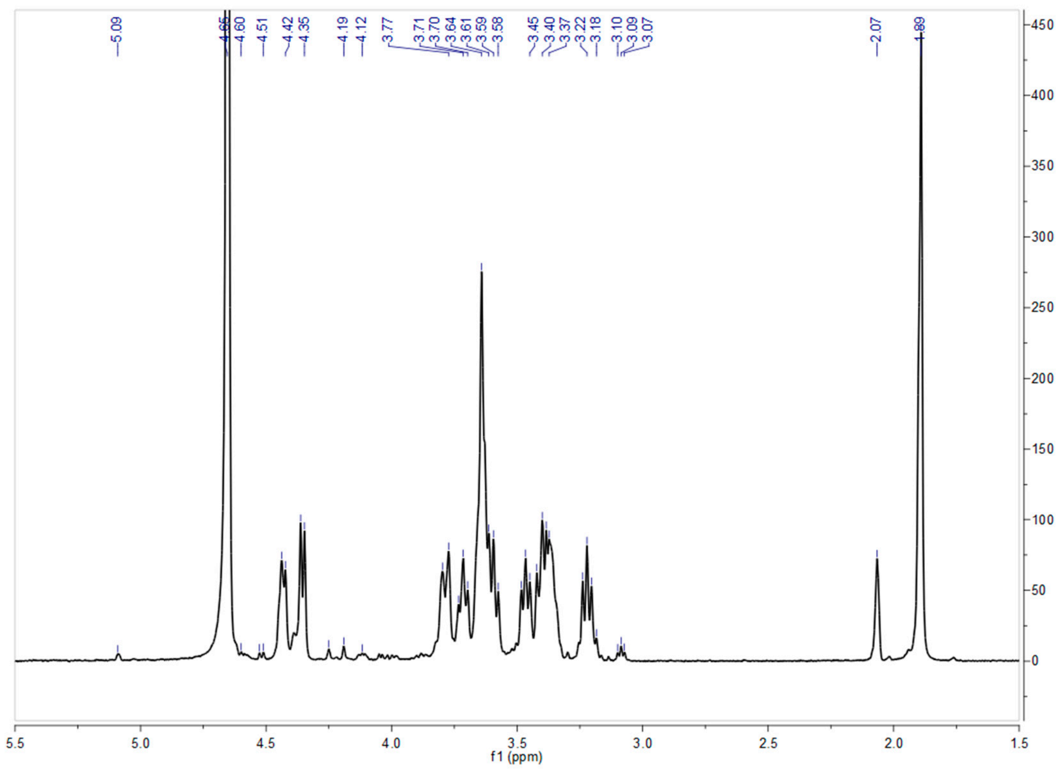

Figure S15. The ^1H NMR spectrum of 11-mer HA

4.The ^1H NMR spectra of HAOs with odd-numbered sugar residues

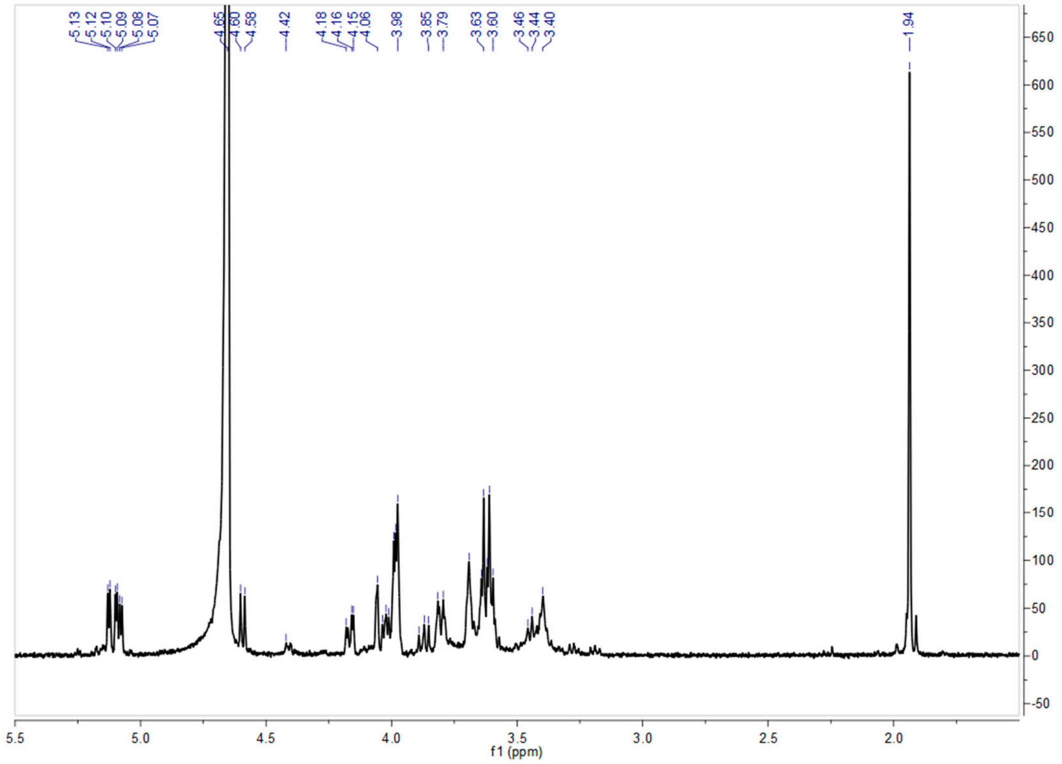

Figure S16. The ^1H NMR spectrum of 2-mer HA

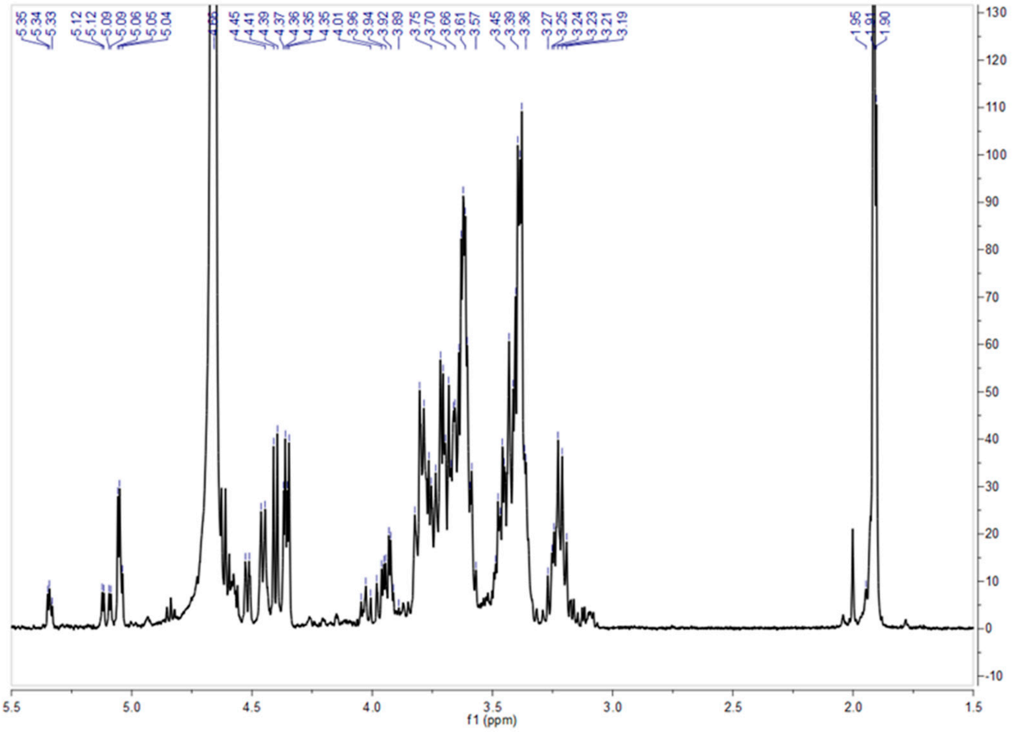

Figure S17. The ¹H NMR spectrum of 4-mer HA

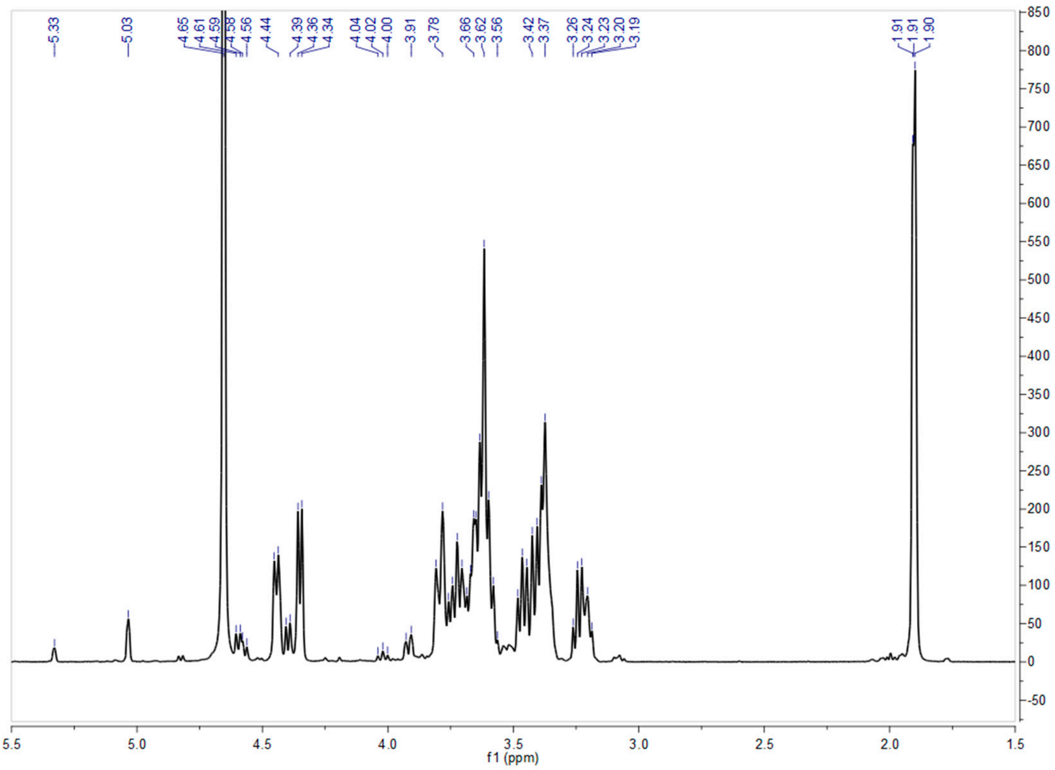

Figure S18. The ¹H NMR spectrum of 6-mer HA

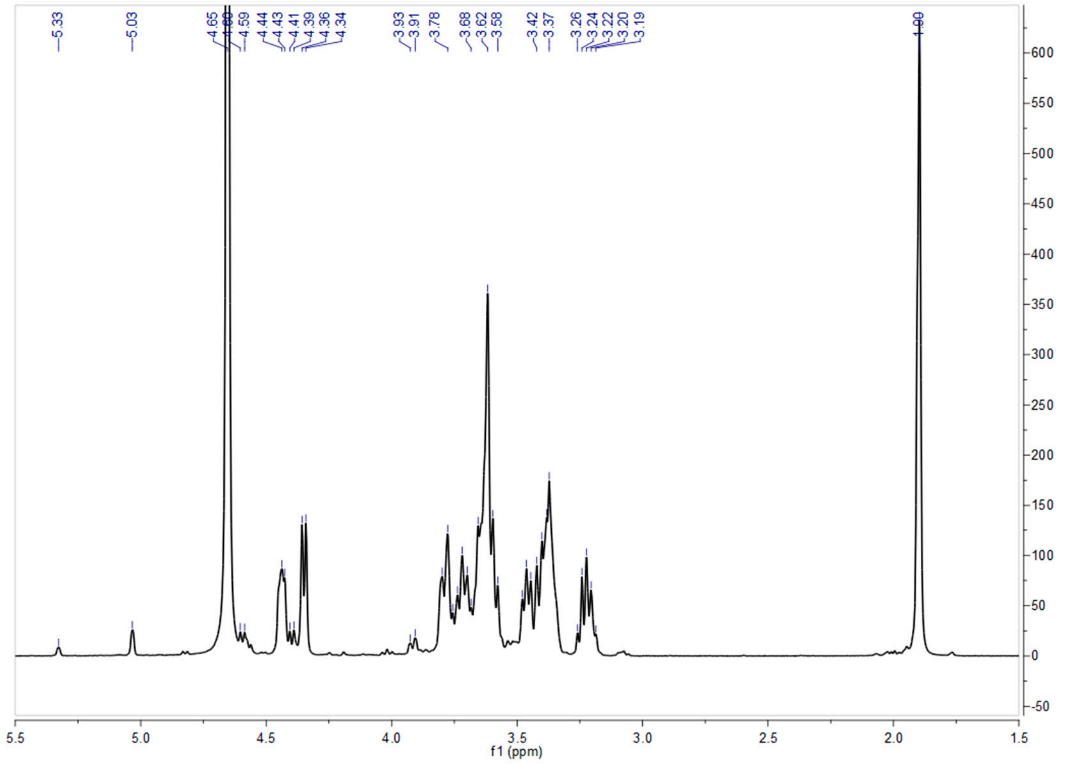

Figure S19. The ¹H NMR spectrum of 8-mer HA

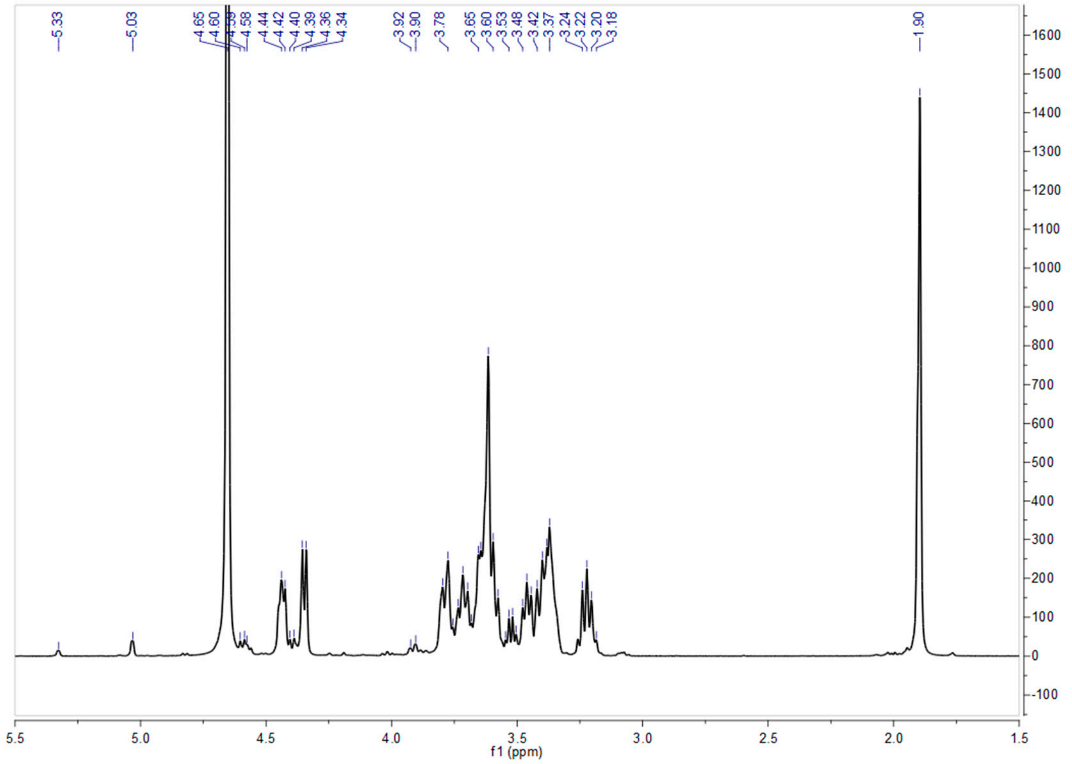

Figureure.S20. The ¹H NMR spectrum of 10-mer HA
